# Preoperative NLR and INR, and intraoperative cold ischemia time as predictors of short-term mortality risk in paediatric liver transplantation: a LASSO-cox model study

**DOI:** 10.1080/07853890.2026.2703891

**Published:** 2026-07-28

**Authors:** Quanhai Zhang, Haifeng Tang, Jie Zhang, Jiajun Weng, Wei Liu, Xiaoke Dai, Mingman Zhang

**Affiliations:** ^a^ Department of Hepatobiliary Surgery, Children’s Hospital of Chongqing Medical University, National Clinical Research Center for Children and Adolescents’ Health and Diseases, Ministry of Education Key Laboratory of Child Development and Disorders, Chongqing Key Laboratory of Pediatric Metabolism and Inflammatory Diseases, Chongqing, China; ^b^ Department of Anesthesiology, Children’s Hospital of Chongqing Medical University, National Clinical Research Center for Children and Adolescents’ Health and Diseases, Ministry of Education Key Laboratory of Child Development and Disorders, Chongqing Key Laboratory of Pediatric Metabolism and Inflammatory Diseases, Chongqing, China

**Keywords:** Paediatric liver transplantation, mortality risk, neutrophil-to-lymphocyte ratio, international normalized ratio, cold ischemia time

## Abstract

**Objectives:**

Liver transplantation (LT) is an effective treatment for end-stage liver disease in children, but precise risk stratification tools for postoperative mortality remain needed.

**Methods:**

We retrospectively analyzed 383 pediatric LT recipients, categorized into death (*n* =31) and survival (*n* =352) groups. Variables were screened via LASSO regression, and independent risk factors were identified using multivariate Cox regression to construct a nomogram. Internal validation employed bootstrap resampling (1,000 iterations). Discrimination and calibration were assessed using Harrell’s C-index, calibration curves, and Brier scores. Predictive performance was compared with Pediatric End-Stage Liver Disease (PELD) and Child-Pugh scores using time-dependent AUC. Decision curve analysis (DCA) evaluated clinical net benefit.

**Results:**

Multivariate Cox regression identified preoperative neutrophil-to-lymphocyte ratio (H R=1.125, 95%CI:1.058–1.197, *p* < 0.001), preoperative international normalized ratio (H R=1.728, 95%CI:1.430–2.087, *p* < 0.001), and cold ischemia time (HR=1.003, 95%CI:1.002–1.005, *p* < 0.001) as independent risk factors. The nomogram achieved a bootstrap-corrected C-index of 0.707 (95%CI:0.607–0.816). Calibration curves showed excellent agreement at 1, 3, and 12 months postoperatively. Time-dependent AUCs at 1 month (0.944), 3 months (0.874), and 12 months (0.774) significantly outperformed PELD and Child-Pugh scores (all *p* < 0.05). High-risk patients exhibited significantly inferior long-term survival (*p* < 0.0001). DCA confirmed positive net benefit.

**Conclusions:**

This nomogram, incorporating preoperative NLR, INR, and cold ischemia time, effectively predicts short- and medium-term mortality after pediatric LT, outperforming conventional scores and offering a reliable tool for individualized risk assessment.

## Introduction

Liver transplantation (LT) represents the definitive therapeutic intervention for paediatric end-stage liver disease, acute liver failure, and congenital metabolic disorders, significantly improving long-term survival and quality of life in affected children [1,2]. Over the past decade, advances in surgical techniques, optimization of immunosuppressive protocols, and improvements in perioperative intensive care have resulted in 1-year post-transplant survival rates exceeding 90% [3,4]. However, despite continuous improvements in surgical success rates, waiting list mortality remains unacceptably high, with overall mortality approximately 6% and approaching 20% in children under 1 year of age [5,6]. This sobering reality underscores the urgent need for accurate identification of high-risk patients, optimization of transplant timing decisions, and establishment of precise postoperative risk stratification management systems.

Early postoperative mortality represents a major bottleneck constraining the efficacy of paediatric LT, involving multifactorial mechanisms including recipient preoperative baseline status, graft quality, surgical technical factors, and early postoperative complications [7,8]. Previous studies have demonstrated that recipient age, nutritional status, severity of underlying disease, and comorbidity burden are closely associated with postoperative outcomes [9,10]. Young recipients, particularly infants, face heightened technical challenges and mortality risks due to immature immune system development, poor surgical tolerance, and difficulties in graft volume matching [11,12]. Furthermore, patients with severe preoperative coagulopathy or infectious complications exhibit significantly elevated rates of postoperative multiple organ failure [7,8].

In current clinical practice, the Paediatric End-Stage Liver Disease (PELD) score and Child-Pugh score are commonly employed tools for prioritizing liver transplantation [13,14]. However, the PELD score, based primarily on serum bilirubin, albumin, INR, age, and growth failure parameters, was originally designed to predict waitlist mortality in chronic liver disease rather than postoperative short-term survival outcomes [15,16]. Multiple validation studies have demonstrated limited discriminatory capacity of the PELD score in predicting early postoperative complications and mortality, with receiver operating characteristic area under the curve (AUC) values frequently below 0.70 [15,17]. Although the Child-Pugh score is widely applied for cirrhosis severity stratification, its subjective nature, fixed indicator weighting, and insufficient validation in paediatric populations limit its applicability [18,19]. A shared limitation of these conventional models is their failure to integrate critical variables such as systemic inflammatory markers and organ preservation quality, resulting in inadequate individualized predictive capability [20].

In recent years, the role of inflammatory-immune imbalance in transplant outcomes has garnered increasing attention. The neutrophil-to-lymphocyte ratio (NLR), as a simple, economical, and reproducible derivative indicator from complete blood count, objectively reflects the balance between systemic inflammation and immunosuppression. In adult liver transplantation, elevated preoperative NLR has been independently associated with postoperative acute kidney injury, infectious complications, and early mortality [21,22]. The underlying mechanisms may involve endothelial damage induced by pro-inflammatory mediators released from neutrophils, as well as cellular immune dysfunction reflected by lymphocytopenia.

Prolonged international normalized ratio (INR) not only reflects the severity of impaired hepatic synthetic function but also indicates portal hypertension-related coagulation factor consumption and activation of the fibrinolytic system [23]. In paediatric patients with acute liver failure, INR serves as a core parameter for assessing disease severity and transplant urgency [24]. Studies have shown that preoperative INR > 2.5 is closely associated with increased postoperative haemorrhagic complications and transfusion requirements [25]. However, the independent association between INR and postoperative mortality in paediatric LT remains to be validated in large-scale cohorts.

Organ procurement and preservation quality represents another critical determinant of transplant outcomes. Cold ischemia time (CIT), as a quantitative indicator of ischemia-reperfusion injury experienced by the graft, is directly associated with hepatocyte apoptosis, sinusoidal endothelial cell damage, and microcirculatory disturbances [26,27]. Adult LT studies have confirmed that CIT exceeding 8–12 h demonstrates a linear positive correlation with postoperative primary graft dysfunction and early graft failure risks [28]. In pediatric LT, due to smaller graft volumes and higher metabolic demands, the critical threshold and clinical impact of CIT may exhibit age-specific variations.

In summary, significant methodological limitations and clinical gaps persist in the field of paediatric LT postoperative mortality risk prediction. The present study aims to systematically integrate preoperative inflammatory markers (NLR), coagulation parameters (INR), and intraoperative organ preservation variables (cold ischemia time) through LASSO-Cox regression analysis to construct and internally validate a nomogram prediction model for 1-month, 3-month, and 12-month postoperative mortality risk following paediatric LT, thereby providing a reliable and user-friendly evidence-based tool for clinical risk stratification and individualized therapeutic decision-making.

## Patients and methods

This retrospective cohort study was conducted in accordance with the Declaration of Helsinki and was approved by the Ethics Committee of the Children’s Hospital Affiliated to Chongqing Medical University [Approval No. (2025) Lun Shen (Lin Yan) No. (78)]. The requirement for informed consent was waived due to the retrospective nature of the study. We hereby declare that none of the organs/tissues were procured from executed prisoners. All organs were procured after informed consent was obtained from the donors or their legal representatives. All 383 patients in the final analytic cohort had complete data for the variables under study, and no statistical imputation was required.

### Study design and patient cohort

This retrospective cohort study included pediatric patients (age < 18 years) who underwent their first liver transplantation at the Department of Hepatobiliary Surgery, Children’s Hospital of Chongqing Medical University, between April 2017 and December 2024. Clinical and follow‑up data were collected from the electronic medical record system and a dedicated transplantation database. A total of 383 patients with complete data were enrolled in the final analysis. Inclusion criteria were: (1) age < 18 years; (2) first-time liver transplantation; (3) complete clinical and follow-up data. Exclusion criteria included: (1) combined multi-organ transplantation; (2) re-transplantation; (3) severe missing data.

### Data collection and definitions

Study variables were retrospectively extracted from the electronic medical record system of the Children’s Hospital of Chongqing Medical University, primarily encompassing the following domains: (1) recipient demographic characteristics and end-stage liver disease model scores; (2) donor characteristics and types; (3) preoperative laboratory parameters (complete blood count, hepatic and renal function, coagulation profile, neutrophil-to-lymphocyte ratio, etc.); (4) intraoperative parameters (cold ischemia time, warm ischemia time, anhepatic phase, intraoperative blood loss, etc.).

Preoperative hepatic functional reserve and disease severity were assessed using the Child-Pugh score and the Pediatric End-Stage Liver Disease (PELD) score. The Child-Pugh score was calculated and graded (Class A: 5–6 points; Class B: 7–9 points; Class C: 10–15 points) according to established criteria [29]. The PELD score was calculated using the formula: 4.80 × ln[total bilirubin (mg/dL)] + 6.87 × ln[INR] + 4.37 × ln[albumin (g/dL)] [30].

### Statistical analysis

Data were described as median (interquartile range, IQR) and frequency (percentage). Group comparisons were performed using the Mann-Whitney U test, Chi-square test, or Fisher’s exact test, as appropriate. To screen risk factors and construct the prediction model, univariate Cox regression analysis was first performed for all variables, followed by dimensionality reduction using LASSO (Least Absolute Shrinkage and Selection Operator) regression (10-fold cross-validation, lambda.1se criterion). Variables selected by LASSO were subsequently incorporated into multivariate Cox proportional hazards regression to identify final independent risk factors. A nomogram model was constructed based on the independent predictors and internally validated using bootstrap resampling (1,000 iterations). Model discrimination was assessed using Harrell’s C-index, while calibration was evaluated using calibration curves and Brier scores. Time-dependent receiver operating characteristic (time-ROC) analysis was employed to calculate the area under the curve (AUC) at different time points (1 month, 3 months, and 12 months), comparing the performance against PELD and Child-Pugh scores; differences in AUC values were compared using time-dependent comparison tests. Patients were stratified into low-risk and high-risk groups according to the total nomogram scores, and survival curves were plotted using the Kaplan-Meier method; intergroup differences were compared using the Log-rank test. Decision Curve Analysis (DCA) was performed to evaluate the clinical net benefit of the model. A two-sided P value < 0.05 was considered statistically significant. All statistical analyses were performed using R version 4.5.1.

## Results

A total of 383 paediatric liver transplant recipients were enrolled in this study, including 31 patients (8.1%) in the death group and 352 patients (91.9%) in the survival group (Tables 1–3). Baseline comparisons between the two groups revealed that recipients in the death group were older (215.00 vs. 174.00 months, *p* = 0.004), with significantly lower preoperative platelet count, lymphocyte count, and lymphocyte percentage, and higher neutrophil-to-lymphocyte ratio (NLR) (all *p* < 0.05). The death group exhibited elevated creatinine, prothrombin time, and INR levels, whereas albumin levels were decreased (all *p* < 0.05). Significant differences were observed in diagnostic distribution between groups (*p* < 0.001), with a lower proportion of biliary atresia in the death group compared with the survival group (61.3% vs. 83.5%). Regarding donor characteristics, the death group had a higher proportion of deceased donor liver transplantation (DDLT) (41.9% vs. 15.3%, *p* < 0.001), with a trend toward older donor age (*p* = 0.069). Intraoperative parameters showed that cold ischemia time, anhepatic phase, portal perfusion to hepatic artery anastomosis time, and intraoperative blood loss were all significantly higher in the death group (all *p* < 0.05), whereas the proportion of graft type (deceased donor whole liver/split liver) was higher in the death group (*p* = 0.001). No significant differences were observed between the two groups in sex, height, weight, PELD score, graft weight, or GRWR (*p* > 0.05).

**Table 1.** Baseline characteristics and intraoperative parameters of paediatrics liver transplant recipients: Death group vs. survival group.

Variable	Total(*n* = 383)	Death Group (*n* = 31)	Survival Group (*n* = 352)	P-value
Demographics				
Age (months)	177.00 (152.00, 257.50)	215.00 (183.00, 1081.00)	174.00 (149.00, 249.25)	**0.004**
Gender				0.54
Male	187 (48.8%)	13 (41.9%)	174 (49.4%)	–
Female	196 (51.2%)	18 (58.1%)	178 (50.6%)	–
Height (cm)	63.00 (60.00, 68.00)	64.00 (60.00, 88.50)	63.00 (60.00, 67.00)	0.659
Weight (kg)	6.50 (5.90, 8.00)	6.80 (5.50, 11.50)	6.50 (5.90, 8.00)	0.711
BMI (kg/m²)	16.39 (15.14, 17.64)	15.85 (14.72, 17.15)	16.39 (15.19, 17.64)	0.324
Disease severity				
PELD	17.33 (9.85, 23.63)	18.27 (13.33, 27.87)	17.20 (9.56, 23.20)	0.304
Child-Pugh	9.00 (7.00, 11.00)	8.00 (7.00, 11.00)	9.00 (7.00, 11.00)	0.657
History of Kasai procedure				1
Yes	45 (11.7%)	4 (12.9%)	41 (11.6%)	–
No	338 (88.3%)	27 (87.1%)	311 (88.4%)	–
Diagnosis				**<0.001**
Biliary atresia (BA)	313 (81.7)	294 (83.5)	19 (61.3)	–
Wilson’s disease	7 (1.8)	6 (1.7)	1 (3.2)	–
Caroli disease	5 (1.3)	4 (1.1)	1 (3.2)	–
Congenital biliary/intrahepatic structural abnormalities	8 (2.1)	7 (2.0)	1 (3.2)	–
Alagille syndrome	5 (1.3)	3 (0.9)	2 (6.5)	–
PFIC3	5 (1.3)	4 (1.1)	1 (3.2)	–
Liver cirrhosis	9 (2.3)	8 (2.3)	1 (3.2)	–
Urea cycle disorder	6 (1.6)	6 (1.7)	0 (0.0)	–
Fulminant liver failure	4 (1.0)	2 (0.6)	2 (6.5)	–
Cavernous transformation of portal vein	6 (1.6)	6 (1.7)	0 (0.0)	–
Glycogen storage disease	2 (0.5)	2 (0.6)	0 (0.0)	–
Portal vascular anomalies	3 (0.8)	3 (0.9)	0 (0.0)	–
Hepatolenticular degeneration	1 (0.3)	1 (0.3)	0 (0.0)	–
Liver tumor and proliferative lesions	3 (0.8)	2 (0.6)	1 (3.2)	–
Autoimmune/overlap liver disease	2 (0.5)	0 (0.0)	2 (6.5)	–
Hepatic echinococcosis (with venous invasion)	1 (0.3)	1 (0.3)	0 (0.0)	–
Maple syrup urine disease	1 (0.3)	1 (0.3)	0 (0.0)	–
Preoperative labs				
WBC (×10⁹/L)	12.97 (8.41, 17.56)	12.18 (6.65, 15.61)	13.17 (8.59, 17.64)	0.394
PLT (×10⁹/L)	235.00 (154.00, 347.00)	183.00 (102.00, 280.50)	240.00 (157.00, 352.50)	**0.023**
RBC (×10¹²/L)	3.65 (3.28, 4.06)	3.61 (3.13, 3.78)	3.65 (3.29, 4.08)	0.06
Hb (g/L)	101.00 (90.50, 112.00)	98.00 (83.50, 109.50)	101.00 (91.00, 113.00)	0.209
LYM (×10⁹/L)	6.91 (3.67, 9.98)	5.59 (1.59, 8.45)	7.21 (3.80, 10.12)	**0.025**
NE (×10⁹/L)	4.29 (2.87, 5.93)	4.95 (3.02, 7.05)	4.25 (2.87, 5.82)	0.222
E (×10⁹/L)	0.24 (0.13, 0.41)	0.17 (0.10, 0.30)	0.25 (0.13, 0.43)	0.071
NLR	0.70 (0.42, 1.21)	0.94 (0.70, 1.58)	0.67 (0.41, 1.21)	**0.009**
PLR	36.67 (26.26, 52.32)	41.29 (29.43, 52.99)	36.39 (25.91, 52.15)	0.472
LYM%	51.20 (35.80, 64.20)	36.00 (0.58, 53.00)	52.20 (36.68, 65.00)	**0.002**
NE%	33.20 (22.85, 47.85)	36.70 (0.65, 46.00)	33.05 (23.00, 47.92)	0.651
E%	2.00 (1.00, 3.00)	0.90 (0.01, 2.75)	2.00 (1.00, 3.00)	**0.018**
TB (μmol/L)	254.40 (136.25, 335.35)	230.20 (19.90, 352.45)	255.25 (148.18, 335.12)	0.53
TP(g/L)	36.50 (31.65, 42.50)	35.80 (29.35, 42.55)	36.60 (31.98, 42.42)	0.533
ALT (U/L)	196.00 (105.50, 303.50)	177.00 (70.25, 281.35)	196.00 (107.75, 304.75)	0.382
AST (U/L)	340.00 (180.50, 509.50)	302.00 (106.60, 435.15)	345.70 (181.75, 527.50)	0.309
Cr (μmol/L)	16.00 (13.35, 21.80)	19.00 (15.00, 33.40)	16.00 (13.30, 21.00)	**0.014**
PT (s)	14.50 (12.40, 17.90)	17.00 (13.05, 23.20)	14.50 (12.30, 17.80)	**0.036**
INR	1.26 (1.08, 1.62)	1.46 (1.13, 2.44)	1.26 (1.08, 1.59)	**0.02**
ALB (g/L)	36.20 (31.15, 42.00)	32.40 (25.60, 38.35)	36.45 (31.60, 42.10)	**0.008**
Blood ammonia (μmol/L)	47.90 (34.90, 60.60)	41.40 (31.83, 53.00)	48.40 (34.90, 60.60)	0.206

Note: Data are presented as median (IQR) or number (%).

PELD: Pediatric End-Stage Liver Disease; PFIC3: progressive familial intrahepatic cholestasis type 3; WBC: white blood cell; PLT: platelet; RBC: red blood cell; Hb: hemoglobin; LYM: lymphocyte; NE: neutrophil; E: eosinophil; NLR: neutrophil-to-lymphocyte ratio; PLR: platelet-to-lymphocyte ratio; TB: total bilirubin; TP: total protein; ALT: alanine aminotransferase; AST: aspartate aminotransferase; Cr: creatinine; PT: prothrombin time; INR: international normalized ratio; ALB: albumin.

Bold values indicate statistical significance (*p* < 0.05).

**Table 2.** Donor baseline characteristics.

Variable	Total (*n* = 383)	Death group (*n* = 31)	Survival group (*n* = 352)	*p*-value
Donor gender				0.245
Male	178 (46.5%)	18 (58.1%)	160 (45.5%)	–
Female	205 (53.5%)	13 (41.9%)	192 (54.5%)	–
Donor age (years)	28.42 (24.83, 32.79)	28.00 (20.38, 31.25)	28.62 (25.04, 33.29)	**0.069**
Donor height (cm)	161.00 (155.00, 168.00)	162.00 (151.00, 169.50)	161.00 (155.00, 168.00)	0.632
Donor weight (kg)	60.00 (52.00, 67.75)	59.10 (54.00, 67.50)	60.00 (51.75, 67.62)	0.601
Donor BMI (kg/m²)	22.83 (20.37, 24.97)	22.86 (19.37, 25.25)	22.81 (20.56, 24.94)	0.707
Donor type				**<0.001**
LDLT (2)	316 (82.5%)	18 (58.1%)	298 (84.7%)	–
DDLT (4)	67 (17.5%)	13 (41.9%)	54 (15.3%)	–
Graft steatosis				0.358
None (0)	155 (40.6%)	16 (53.3%)	139 (39.5%)	–
Microvesicular (1)	132 (34.6%)	7 (23.3%)	125 (35.5%)	–
Macrovesicular <30% (2)	88 (23.0%)	6 (20.0%)	82 (23.3%)	–
Macrovesicular 30%–60% (3)	7 (1.8%)	6 (1.7%)	1 (3.3%)	–
Transplant type				**0.001**
Deceased whole liver	38 (9.9%)	6 (19.4%)	32 (9.1%)	–
Deceased reduced/split liver	29 (7.6%)	7 (22.6%)	22 (6.2%)	–
Living donor	316 (82.5%)	18 (58.1%)	298 (84.7%)	–

Note: Data are presented as median (IQR) or number (%).

BMI: body mass index; DDLT: deceased donor liver transplantation; LDLT: living donor liver transplantation.

Bold values indicate statistical significance (*p* < 0.05).

**Table 3.** Intraoperative parameters.

Variable	Total (*n* = 383)	Death group (*n* = 31)	Survival group (*n* = 352)	*p*-value
Warm ischemia time (min)	1.62 (1.17, 2.32)	1.62 (0.93, 3.00)	1.62 (1.18, 2.29)	0.689
Cold ischemia time (min)	87.00 (73.00, 143.50)	161.00 (102.00, 378.50)	85.00 (72.00, 131.50)	**<0.001**
Anhepatic phase (min)	52.00 (45.00, 63.00)	64.00 (54.00, 78.00)	51.00 (45.00, 62.00)	**<0.001**
Time from portal perfusion to hepatic artery anastomosis (min)	70.00 (60.00, 86.00)	85.00 (61.50, 114.00)	69.50 (60.00, 84.25)	**0.005**
Total operation time (h)	7.67 (6.92, 8.68)	8.33 (7.06, 9.89)	7.67 (6.92, 8.58)	0.06
Intraoperative blood loss (ml)	350.00 (250.00, 600.00)	400.00 (300.00, 1125.00)	350.00 (250.00, 550.00)	**0.026**
Graft weight (g)	250.00 (220.00, 300.00)	250.00 (207.50, 340.00)	250.00 (220.00, 300.00)	0.82
GRWR%	3.67 (2.93, 4.35)	3.57 (2.93, 4.67)	3.67 (2.93, 4.33)	0.994
Transplant type				**0.001**
Deceased donor, whole liver	38 (9.9%)	6 (19.4%)	32 (9.1%)	–
Deceased donor, split liver	29 (7.6%)	7 (22.6%)	22 (6.2%)	–
Living donor	316 (82.5%)	18 (58.1%)	298 (84.7%)	–

Note: Data are presented as median (IQR) or number (%).

GRWR: Graft-to-Recipient Weight Ratio.

Bold values indicate statistical significance (*p* < 0.05).

### Independent risk factors for postoperative mortality following paediatric liver transplantation identified by LASSO regression

Candidate predictive variables with *p* < 0.1 from univariate Cox regression analysis were selected. To further control for overfitting and multicollinearity, LASSO regression combined with 10-fold cross-validation was employed for variable selection. Variables with non-zero coefficients from LASSO regression were subsequently incorporated into the multivariate Cox regression model (Tables 4–6). Multivariate Cox regression analysis ultimately identified only preoperative NLR (HR = 1.125, 95% CI: 1.058–1.197, *p* < 0.001), preoperative INR (HR = 1.728, 95% CI: 1.430–2.087, *p* < 0.001), and intraoperative cold ischemia time (HR = 1.003 per minute increase, 95% CI: 1.002–1.005, *p* < 0.001) as independent risk factors. Although the HR is close to 1, reflecting a modest effect per minute, prolonged cold ischemia (e.g. >600 min) can cumulatively increase risk by approximately 20%, supporting its clinical relevance.

**Table 4.** Univariate and multivariate Cox regression analyses for predicting mortality after paediatrics liver transplantation.

Variable	Univariate analysis HR (95% CI)	*p*-value	Multivariate analysis HR (95% CI)	*p*-value
Demographics				
Age (months)	1.000 (1.000-1.000)	0.127	–	–
Gender (0 = Male, 1 = Female)	1.389 (0.680–2.838)	0.367	–	–
Height (cm)	1.008 (0.996–1.020)	0.204	–	–
Weight (kg)	1.021 (0.988–1.056)	0.218	–	–
BMI (kg/m²)	0.935 (0.802–1.090)	0.387	–	–
Disease severity	.			
PELD	1.014 (0.991–1.037)	0.225	–	–
Child-Pugh	0.964 (0.873–1.065)	0.471	–	–
Prior Kasai procedure (1 = Yes, 0 = No)	1.053 (0.367–3.017)	0.924	–	–
Preoperative labs				
WBC (×10⁹/L)	0.977 (0.926–1.031)	0.399	–	–
PLT (×10⁹/L)	0.997 (0.994–1.000)	**0.033**	–	–
RBC (×10¹²/L)	0.561 (0.322–0.977)	**0.041**	–	–
Hb (g/L)	1.006 (0.996–1.015)	0.235	–	–
LYM (×10⁹/L)	0.911 (0.831–0.999)	**0.047**	–	–
NE (×10⁹/L)	1.034 (0.967–1.106)	0.322	–	–
E (×10⁹/L)	0.988 (0.911–1.072)	0.774	–	–
NLR	1.103 (1.059–1.149)	**<0.001**	1.125 (1.058–1.197)	**<0.001**
PLR	1.007 (1.000–1.014)	0.052	–	–
LYM%	0.975 (0.961–0.988)	**<0.001**	–	–
NE%	0.995 (0.978–1.013)	0.588	–	–
E%	0.724 (0.548–0.958)	**0.024**	–	–
TB (μmol/L)	0.999 (0.996–1.001)	0.305	–	–
TP(g/L)	1.034 (1.022–1.046)	**<0.001**	–	–
ALT (U/L)	1.001 (0.999–1.002)	0.215	–	–
AST (U/L)	1.000 (0.999–1.001)	0.993	–	–
Cr (μmol/L)	1.007 (1.004–1.010)	**<0.001**	–	–
PT (s)	1.049 (1.016–1.083)	**0.003**	–	–
INR	1.601 (1.375–1.865)	**<0.001**	1.728 (1.430–2.087)	**<0.001**
ALB(g/L)	0.894 (0.858–0.932)	**<0.001**	–	–
Blood ammonia (μmol/L)	0.992 (0.975–1.009)	0.345	–	–

Note: Data are presented as median (IQR) or number (%).

PELD: Paediatric End-stage Liver Disease score; WBC: White blood cell count; PLT: Platelet count; RBC: Red blood cell count; Hb: Hemoglobin; LYM: Lymphocyte count; NE: Neutrophil count; E: Eosinophil count; NLR: Neutrophil-to-lymphocyte ratio; PLR: Platelet-to-lymphocyte ratio; LYM%: Lymphocyte percentage; NE%: Neutrophil percentage; E%: Eosinophil percentage; TB: Total bilirubin; TP: Total Protein; ALT: Alanine aminotransferase; AST: Aspartate aminotransferase; Cr: Creatinine; PT: Prothrombin time; INR: International normalized ratio.

Bold values indicate statistical significance (*p* < 0.05).

**Table 5.** Donor baseline characteristics.

Variable	Univariate analysis HR (95% CI)	*p*-value	Multivariate analysis HR (95% CI)	*p*-value
Donor gender (1 = Male, 2 = Female)	0.617 (0.302–1.259)	0.185	–	–
Donor age (years)	0.965 (0.934–0.998)	**0.035**	–	–
Donor height (cm)	0.987 (0.976–0.999)	**0.028**	–	–
Donor weight (kg)	0.985 (0.966–1.005)	0.133	–	–
Donor BMI (kg/m²)	0.958 (0.869–1.055)	0.384	–	–
Donor type			–	–
DDLT (vs. LDLT)	3.692 (1.808–7.537)	<0.001	–	–
Transplant type			–	–
Deceased donor, whole liver	3.692 (1.808–7.537)	**<0.001**	–	–
Deceased donor, split liver	1.612 (0.541–4.797)	0.391	–	–
Living donor	0.340 (0.135–0.858)	**0.022**	–	–
Graft steatosis			–	–
Microvesicular (vs. none)	0.511 (0.210–1.242)	0.138	–	–
Macrovesicular <30% (vs. none)	0.655 (0.256–1.674)	0.377	–	–
Macrovesicular 30%–60% (vs. none)	1.460 (0.193–11.030)	0.714	–	–

Note: Data are presented as median (IQR) or number (%).

BMI: body mass index; DDLT: deceased donor liver transplantation; LDLT: living donor liver transplantation.

Bold values indicate statistical significance (*p* < 0.05).

**Table 6.** Intraoperative parameters.

Variable	Univariate analysis HR (95% CI)	*p*-value	Multivariate analysis HR (95% CI)	*p*-value
Warm ischemia time (min)	1.024 (0.863–1.214)	0.788	–	–
Cold ischemia time (min)	1.004 (1.002–1.005)	**<0.001**	1.003 (1.002–1.005)	**<0.001**
Anhepatic phase (min)	1.010 (1.003–1.016)	**0.004**	–	–
Time from portal perfusion to hepatic artery anastomosis (min)	1.025 (1.012–1.037)	**<0.001**	–	–
Total operation time (h)	1.356 (1.162–1.584)	**<0.001**	–	–
Intraoperative blood loss (mL)	1.000 (1.000–1.001)	0.269	–	–
Graft weight (g)	1.001 (1.000–1.002)	0.086	–	–
GRWR%	1.008 (0.752–1.351)	0.958	–	–

Note: Data are presented as median (IQR) or number (%).

GRWR: Graft-to-Recipient Weight Ratio.

Bold values indicate statistical significance (*p* < 0.05).

### Nomogram for predicting postoperative mortality risk in paediatric liver transplantation

**Figure 1.**
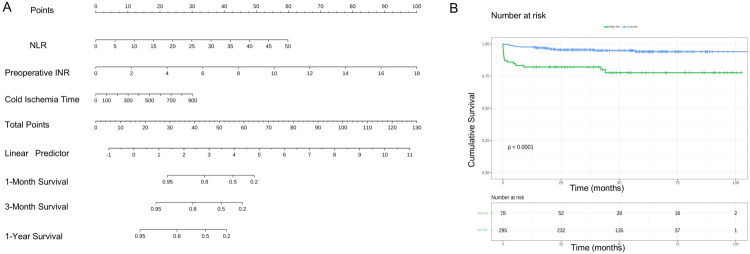
Nomogram for predicting 1-month, 3-month, and 12-month survival after pediatric liver transplantation and risk stratification. (A) Nomogram integrating preoperative NLR, preoperative INR, and cold ischemia time. To use the nomogram, locate each variable value on its axis, draw a vertical line to the ‘Points’ axis to obtain the point score, sum the points, and project the total points onto the ‘Linear Predictor’ and corresponding survival probability axes. (B) Kaplan-Meier survival curves for paediatrics patients stratified into low-risk and high-risk groups based on the optimal cut-off value of the nomogram total score. The number of patients at risk at each time point is shown below the *x*-axis. The log-rank test yielded a *p*-value < 0.0001.

Based on the multivariate Cox regression results, a nomogram model was constructed to predict patient survival probability (Figure 1A). This model incorporated three independent predictive factors: neutrophil-to-lymphocyte ratio, preoperative international normalized ratio, and cold ischemia time. For each patient, individual indicators were assigned corresponding scores based on their specific values on the respective axes. The sum of these scores yielded the total score (0–120 points). According to the total score, the corresponding linear predictor value (−1 to 9 points) could be read from the ‘Linear Predictor’ axis below. Finally, based on this linear predictor value, the respective probability estimates (0.95, 0.80, 0.50, or 0.20) could be obtained from the 1-month, 3-month, and 1-year survival probability axes.

### Nomogram-derived risk stratification and Kaplan‑Meier survival estimates

Based on the total nomogram score, using the median score as the cutoff (Youden index from time-dependent ROC at 3 months), the cohort was stratified into a high‑risk group (*n* = 78) and a low‑risk group (*n* = 295). Kaplan‑Meier analysis revealed a significant divergence in survival trajectories between the two strata (Figure 1B). The low‑risk group maintained a consistently high cumulative survival probability, remaining above 95% at 100 months of follow‑up. In contrast, the high‑risk group experienced a steep decline in survival during the early postoperative period (0–5 months), with cumulative survival falling to approximately 85% by 5 months and further decreasing to approximately 78% at 100 months. The difference in overall survival between the high‑risk and low‑risk groups was statistically significant (log‑rank *p* < 0.0001), confirming the robust discriminatory capacity of the nomogram‑based risk stratification.

### Construction and validation of the prognostic model for postoperative mortality risk following liver transplantation based on LASSO-cox regression

To comprehensively evaluate the performance and generalizability of the final multivariate Cox prediction model (including cold ischemia time, preoperative NLR, and INR), rigorous internal validation was performed using bootstrap resampling (1,000 iterations). Model discrimination was quantified using Harrell’s C-index. The original C-index was 0.709. Following bootstrap correction, the C-index was 0.707 (95% CI: 0.607–0.816).

**Figure 2.**
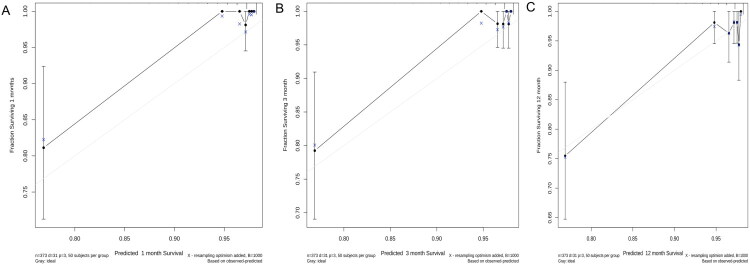
Calibration curves of the nomogram for predicting postoperative survival at 1, 3, and 12 months after paediatrics liver transplantation. (A) Calibration plot for 1‑month survival prediction. The predicted probabilities showed excellent agreement with observed probabilities, with data points closely clustered around the ideal diagonal line. The Brier score was 0.0110, indicating high predictive accuracy. (B) Calibration plot for 3‑month survival prediction. When predicted probabilities ranged from 0.79 to 0.99, observed probabilities stabilized around 0.80, suggesting a slight tendency toward overestimation; overall consistency remained acceptable. The Brier score was 0.0181. (C) Calibration plot for 12‑month survival prediction. Predicted and observed probabilities demonstrated good consistency throughout the range (0.78–1.00), with minor deviations at high predicted probabilities (>0.95). The Brier score at this time point was 0.0352, remaining within a low range and confirming reliable calibration. The model was internally validated using 1,000 bootstrap resamples, yielding a bootstrap‑corrected Harrell’s C‑index of 0.707 (95% CI: 0.607–0.816). Data points represent decile‑grouped patients, and the solid line is a LOESS smooth with 95% confidence intervals shaded in gray.

At 1 month postoperatively (Figure 2A): The bootstrap-corrected calibration curve demonstrated excellent agreement between model-predicted survival probabilities and actual Kaplan-Meier estimated probabilities, with data points clustering tightly around the ideal diagonal line. The Brier score was the lowest at 0.0110, indicating excellent predictive accuracy and calibration precision in the near-term.

At 3 months postoperatively (Figure 2B): The calibration curve showed that when predicted probabilities ranged from 0.79 to 0.99, the corresponding observed probabilities stabilized around 0.80, indicating a slight tendency toward overestimation by the model, yet overall consistency was acceptable. The Brier score increased slightly to 0.0181, with prediction error remaining at a low level.

At 12 months postoperatively (Figure 2C): The calibration curve demonstrated that predicted and actual probabilities maintained good consistency throughout the range (0.78–1.00), although minor deviations existed in the high predicted probability interval (>0.95). The Brier score at this time point was 0.0352, the highest among the three time points, yet still within a low range, confirming that the model maintained reliable calibration in medium-term prediction.

### Comparison of model predictive performance: Time-dependent AUC analysis

**Figure 3.**
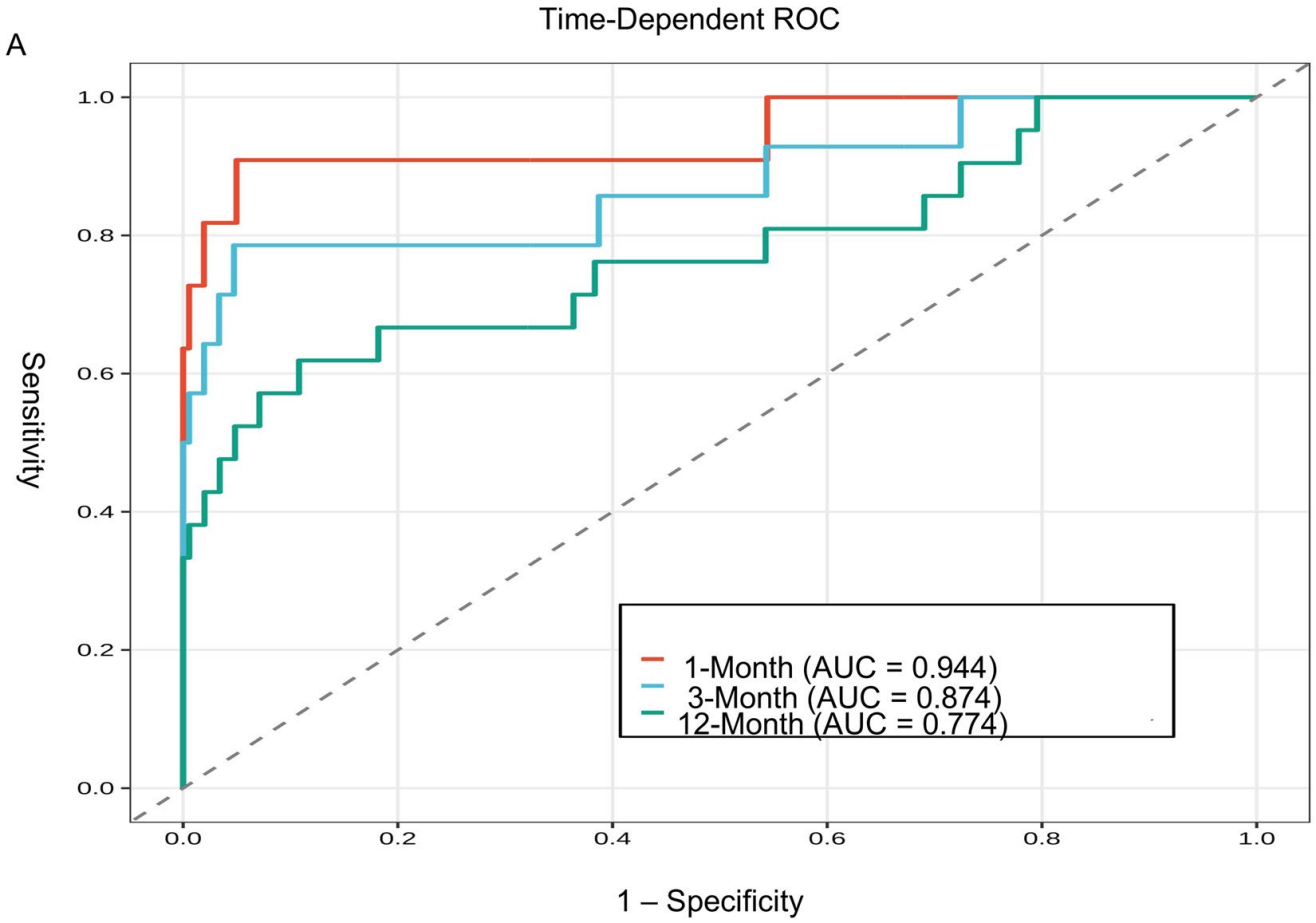
Time-dependent receiver operating characteristic (ROC) curves of the nomogram for predicting postoperative mortality following paediatrics liver transplantation. (A) The ROC curves demonstrate the discriminative performance of the nomogram at three clinically relevant postoperative time points: 1 month (blue line), 3 months (red line), and 12 months (green line). The area under the ROC curve (AUC) was 0.944 at 1 month, 0.874 at 3 months, and 0.774 at 12 months, indicating excellent short-term discrimination and robust medium-term predictive accuracy. The progressive decline in AUC with increasing follow-up duration reflects the anticipated attenuation of baseline covariate effects over time, consistent with the growing influence of post-transplant clinical events on long-term mortality. The diagonal dashed line represents the reference line of no discrimination (AUC = 0.5). Full 95% confidence intervals for each AUC are provided in the corresponding results section.

At 1, 3, and 12 months post‑transplant, 11, 14, and 21 deaths had occurred, respectively. The time‑dependent AUC analysis showed that this study constructed a multivariate Cox proportional hazards model and employed time-dependent area under the receiver operating characteristic curve (time-dependent AUC) to assess model discrimination at different time points (Figure 3A). The newly constructed comprehensive prediction model was compared with two commonly used clinical scoring systems (PELD score and Child-Pugh score) using time-dependent AUC comparison tests.

**Figure 4.**
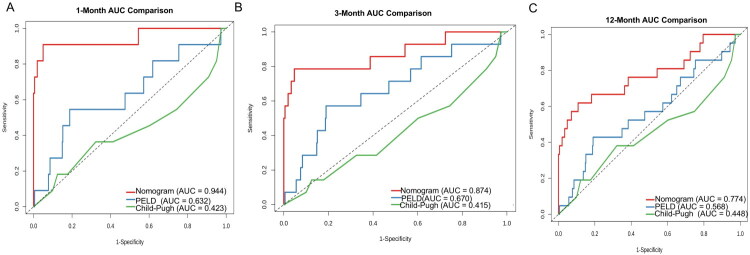
Comparison of receiver operating characteristic (ROC) curves for the Nomogram, PELD score, and Child-Pugh score in predicting [Insert Outcome, e.g. mortality]. (A) Discriminative performance evaluated in the [training cohort/internal validation]. The area under the curve (AUC) was 0.944 for the Nomogram, 0.632 for the Paediatric End-Stage Liver Disease (PELD) score, and 0.423 for the Child-Pugh score. (B) Predictive accuracy for 3-month [outcome]. The Nomogram achieved an AUC of 0.874, demonstrating superior discrimination compared to the PELD score (AUC = 0.670) and the Child-Pugh score (AUC = 0.415). (C) Predictive accuracy for 12-month [outcome]. The Nomogram maintained robust performance with an AUC of 0.774, whereas both the PELD (AUC = 0.568) and Child-Pugh (AUC = 0.448) scores exhibited limited prognostic utility at the extended follow-up. The diagonal reference line represents an AUC of 0.500, indicating no discrimination.

In early (1-month) prediction (Figure 4A), the model achieved an AUC of 0.944, demonstrating excellent discriminatory ability. This AUC was significantly higher than that of the PELD model (AUC = 0.632; AUC difference = 0.425, *p* < 0.001) and the Child-Pugh model (AUC = 0.423; AUC difference = 0.655, *p* < 0.001). No statistically significant difference in predictive performance was observed between the PELD and Child-Pugh models (*p* = 0.208).

At the medium-term (3-month) follow-up time point (Figure 4B), the model maintained significant predictive advantage with an AUC of 0.874. This AUC was significantly superior to the PELD model (AUC = 0.670; AUC difference = 0.368, *p* = 0.001) and the Child-Pugh model (AUC = 0.415; AUC difference = 0.634, *p* < 0.001). The difference between PELD and Child-Pugh models remained statistically non-significant (*p* = 0.099).

At the long-term (12-month) time point, the model achieved an AUC of 0.774 (Figure 4C), maintaining significant predictive superiority over traditional clinical scores. This AUC was significantly higher than the PELD model (AUC = 0.568; AUC difference = 0.253, *p* = 0.028) and the Child-Pugh model (AUC = 0.448; AUC difference = 0.334, *p* = 0.005). The AUC difference between PELD and Child-Pugh models further narrowed and showed no statistical significance (*p* = 0.588).

Decision curve analysis showed that the nomogram provided a positive net benefit over the ‘treat-all’ and ‘treat-none’ strategies only at low threshold probabilities (0%–10% at 1 month, 0%–15% at 3 months, and 0%–3% at 12 months). Beyond these ranges, the net benefit approached zero.

## Discussion

Based on 383 pediatric liver transplantation cases, this study constructed and validated a postoperative mortality risk prediction model comprising three indicators—preoperative neutrophil-to-lymphocyte ratio (NLR), preoperative international normalized ratio (INR), and cold ischemia time—using LASSO-Cox regression methodology. The model demonstrated acceptable discrimination and good calibration, with a bootstrap-corrected C-index of 0.707 (95% CI: 0.607–0.816). This level of discrimination, while moderate, is still significantly superior to that of traditional scores and merits candid discussion: preoperative and intraoperative static variables alone have inherent limitations in predicting long-term survival. The observed decay in time-dependent AUC from 0.944 at 1 month to 0.774 at 12 months further supports this interpretation—early mortality is heavily driven by baseline recipient condition and graft quality, whereas late mortality increasingly depends on post-transplant events not captured here [29], such as acute cellular rejection, infections, immunosuppressive regimen adherence, and biliary complications. Future iterations of the model may achieve improved long-term predictive accuracy by incorporating early postoperative dynamic variables, including delayed graft function, early rejection episodes, and the trajectory of inflammatory markers [30].

This study confirmed that elevated preoperative NLR is an independent risk factor for postoperative mortality (HR = 1.125, 95% CI: 1.058–1.197). Consistent with previous findings in adult liver transplantation regarding the association between inflammatory markers and poor prognosis [28,31], our results extend this relationship to the paediatric setting. Prolonged preoperative INR (HR = 1.728, 95% CI: 1.430–2.087) reflects the severity of pre-existing coagulopathy and the detrimental impact of end-stage liver disease on transplant outcomes [28]. Furthermore, prolonged cold ischemia time (HR = 1.003, 95% CI: 1.002–1.005), as a modifiable intraoperative factor, emphasizes the importance of optimizing organ procurement and transport protocols [32]. Although intraoperative blood loss was significantly higher in the death group on univariate analysis, it was not selected by LASSO regression, likely due to collinearity with cold ischemia time or INR, or limited statistical power given the modest number of events (*n* = 31). Larger prospective studies are needed to clarify its independent role. Notably, this study found no significant association between donor age, weight, and recipient prognosis, suggesting that in paediatric liver transplantation, recipient preoperative condition and organ preservation quality may outweigh donor demographic characteristics in prognostic significance [33,34].

The nomogram model constructed in this study transforms continuous variables into a visual scoring system, enabling individualized survival probability prediction. Risk stratification based on this model effectively distinguished high-risk from low-risk populations, with significantly different long-term survival curves between the two groups (*p* < 0.0001), providing an intuitive reference for clinical decision-making.

Decision curve analysis demonstrated that the nomogram yielded a positive net benefit within low threshold probability ranges—specifically 0%–10% for 1-month, 0%–15% for 3-month, and 0–3% for 12-month mortality—beyond which the net benefit approached that of default strategies [35].

This finding does not indicate a weakness of the model; rather, it defines its appropriate clinical application. In the context of paediatrics liver transplantation, where mortality risk is inherently low given current surgical and perioperative standards [36], the model is best suited to guide decisions regarding intensified postoperative monitoring, such as more frequent laboratory surveillance, earlier imaging, or extended intensive care observation [37]. Conversely, the lack of net benefit at higher threshold probabilities indicates that the model should not be used in isolation to justify high-risk interventions such as urgent re-transplantation or futility determinations. Clarifying these boundaries of clinical utility is essential for the responsible deployment of any prognostic tool.

This study has several limitations. First, the small number of death events (*n* = 31) limited the predictors that could be reliably retained; variables such as liver failure aetiology and intraoperative blood loss showed significant baseline differences between survivors and non‑survivors but were not selected by LASSO, likely due to low statistical power. This may partly explain the moderate C‑index (0.707) and wide confidence intervals. Second, the model captures only pre‑ and intraoperative static variables and omits post‑transplant events that increasingly influence late mortality. Future multicenter prospective studies with larger sample sizes should reassess the independent contribution of primary diagnosis, integrate early postoperative dynamic predictors, and externally validate the refined model.

## Conclusion

The nomogram prediction model developed in this study based on preoperative NLR, preoperative INR, and cold ischemia time demonstrates superior performance to traditional scoring systems in assessing short- and medium-term mortality risk following pediatric liver transplantation. This model provides a reliable tool for individualized risk assessment and perioperative management. Future multi-center prospective studies are needed to externally validate and further refine the model by integrating novel biomarkers and early post-transplant clinical parameters.

## Data Availability

The datasets generated and/or analysed during the current study are not publicly available due to patient privacy and ethical restrictions. However, a de-identified dataset may be made available from the corresponding author on reasonable request and with the approval of the Ethics Committee of the Children’s Hospital Affiliated to Chongqing Medical University.
